# Intakes of Zinc, Potassium, Calcium, and Magnesium of Individuals with Type 2 Diabetes Mellitus and the Relationship with Glycemic Control

**DOI:** 10.3390/nu10121948

**Published:** 2018-12-08

**Authors:** Paula Nascimento Brandão-Lima, Gabrielli Barbosa de Carvalho, Ramara Kadija Fonseca Santos, Beatriz da Cruz Santos, Natalia Lohayne Dias-Vasconcelos, Vivianne de Sousa Rocha, Kiriaque Barra Ferreira Barbosa, Liliane Viana Pires

**Affiliations:** 1 Health Sciences Postgraduate Program, Department of Medicine, Federal University of Sergipe, Rua Cláudio Batista, S/N, Cidade Nova, Aracaju, 49060-108 Sergipe, Brazil; paulanblima@gmail.com (P.N.B.-L.); kiribarra@yahoo.com.br (K.B.F.B.); 2 Nutrition Sciences Postgraduate Program, Department of Nutrition, Federal University of Sergipe, Avenida Marechal Rondon, S/N, Jardim Rosa Elze, São Cristovão, 49100-000 Sergipe, Brazil; gabicarvalho_31@hotmail.com (G.B.d.C.); rkadijanutri@gmail.com (R.K.F.S.); 3 Undergraduate Nutrition Program, Department of Nutrition, Federal University of Sergipe, Avenida Marechal Rondon, S/N, Jardim Rosa Elze, São Cristovão, 49100-000 Sergipe, Brazil; cruz14_bia@outlook.com (B.d.C.S.); diaslohayne@gmail.com (N.L.D.-V.); 4 Department of Nutrition, Federal University of Sergipe, Avenida Governador Marcelo Déda, 13, Centro, Lagarto, 49400-000 Sergipe, Brazil; viviannesrocha@gmail.com

**Keywords:** micronutrients, trace elements, food, glycated hemoglobin A, hyperglycemia

## Abstract

The role of the concomitant intake of zinc, potassium, calcium, and magnesium in the glycemic control of individuals with type 2 diabetes mellitus (T2DM) has not been extensively discussed. We evaluated the relationship between the dietary intake of these micronutrients and glycemic markers in 95 individuals with T2DM (mean age 48.6 ± 8.4 years). Hierarchical grouping analysis was used to divide the individuals into two clusters according to their micronutrient intake, and differences between clusters were statistically assessed. Effects of individual and combination intake of micronutrients on glycated hemoglobin percentage (%HbA1c) were assessed using multiple linear regression and binary logistic regression analysis. We observed a high likelihood of inadequate intake of the four micronutrients. The group with lower micronutrient intake (cluster 1) displayed higher %HbA1c (*p* = 0.006) and triglyceride (*p* = 0.010) levels. High %HbA1c showed an association with cluster 1 (odds ratio (OR) = 3.041, 95% confidence interval (CI) = 1.131; 8.175) and time of T2DM diagnosis (OR = 1.155, 95% CI = 1.043; 1.278). Potassium (β = −0.001, *p* = 0.017) and magnesium (β = −0.007, *p* = 0.015) intakes were inversely associated with %HbA1c. Reduced concomitant intake of the four micronutrients studied proved to be associated with risk of increased %HbA1c in individuals with T2DM, which was particularly predicted by magnesium and potassium intakes.

## 1. Introduction

Type 2 diabetes mellitus (T2DM) is characterized by failures in blood glucose homeostasis and is considered a global public health problem [1]. Chronic hyperglycemia leads to increased oxidative stress and production of proinflammatory cytokines, disrupting insulin signaling pathways, lipid metabolism, protein synthesis, and cell differentiation, and may alter body concentrations of micronutrients [2,3,4,5]. The maintenance of the glycemic control in individuals with T2DM involves strategies for lifestyle changes, drug therapy, and adoption of healthy eating habits [1,2]. A balanced diet with adequate nutrient content can reduce the glycated hemoglobin percentage (%HbA1c) in subjects with T2DM by 0.3–2% [6]. Over the last few years, studies have verified the participation of minerals in the synthesis, secretion, and action of insulin. Among them, the minerals zinc, potassium, calcium, and magnesium are considered essential for the homeostasis of glucose metabolism [5,7,8].

Zinc plays a key role in the insulin biosynthesis as part of the hexameric structure of this hormone, and in the sensitivity to insulin in target tissues through stimulation of insulin receptors [9,10]. Calcium and potassium regulate voltage-dependent channels in pancreatic β-cells, which are essential for insulin exocytosis [11,12]. Magnesium is important for β-cell functioning and acts as a cofactor of many enzymes involved in the glucose metabolism, like tyrosine kinase enzymes, which phosphorylate insulin receptors and trigger the signaling cascade [4,12,13].

Thus, an inadequate intake of these micronutrients may impair the insulin synthesis, secretion, and signaling pathways [4,11,14]. It is possible to observe in the literature studies that associate the low intake and serum concentration of these minerals with risk of T2DM development [2,13,15,16,17,18]. Nevertheless, studies that evaluate the relationship between the dietary intake of these minerals in glycemic control in subjects with established disease are not widely found. In addition, no studies have assessed the relationship of the concomitant intake of these four micronutrients with the glycemic control, insulin biosynthesis, and insulin sensitivity of individuals with T2DM. In this scenario, the present study aimed to evaluate the relationship of the dietary intake of zinc, potassium, calcium, and magnesium with glycemic markers in individuals with T2DM in order to expand the understanding of these relationships in glycemic control.

## 2. Materials and Methods

### 2.1. Study Design and Participants

In this cross-sectional study, to be eligible for enrollment, individuals had to be 19–59 years old and have diagnosis of T2DM. Thus, 102 individuals with T2DM who visited the Family Health Units in the state of Sergipe, Brazil, were evaluated. Exclusion criteria adopted were the use of vitamin–mineral supplements, pregnancy, status as a current smoker, and the presence of the following diseases: rheumatoid arthritis, cancer, chronic renal failure, thyroid dysfunction, and acute infections or inflammatory processes such as influenza and urinary tract infection. The study followed the guidelines of the Declaration of Helsinki and all participants provided written informed consent. The study was approved by the Ethical Committee of the Federal University of Sergipe (Approval number 1.370.831).

Venous blood samples were obtained, after an overnight fast for at least 10 hours, in ethylenediamine tetra-acetic acid (EDTA) tubes for estimation of concentration of glycated hemoglobin (HbA1c) in whole blood, and in gel tubes for the determination of fasting glucose, lipid profile, insulin, and C-peptide in serum. Measurements of anthropometric parameters, body fat percentage, and blood pressure were performed. All the measurements were realized by trained technicians, and the individuals were instructed to avoid physical activity or effort one day before the tests. In combination, dietary intake assessments of macro and micronutrients was performed. Information about socioeconomic conditions, medical history, and lifestyle were obtained using a questionnaire.

### 2.2. Anthropometric Parameters, Bioelectric Impedance and Blood Pressure Measurements

Weight and height measurements were performed to calculate the body mass index (BMI). The BMI was calculated by dividing the body weight value (in kilograms) by the square value of the body height (in meters). The participants were classified as malnourished (BMI < 18.5 kg/m^2^), normal weight (BMI 18.5–24.9 kg/m^2^) or overweight/obese (BMI ≥ 25 kg/m^2^), assessed according to the cut-off values proposed by the World Health Organization [19]. Waist circumference was measured with a non-extendable tape positioned at the midpoint between the last floating rib and the top of the iliac crest. The cut-off point used for waist circumference was ≥80 cm for women and ≥94 cm for men [20].

Measurement of body fat percentage was performed using the BIA 310 Bioimpedance Analyzer (Biodynamics Corporation, Shoreline, WA, USA), following the manufacturer’s instructions. Blood pressure was measured using aneroid sphygmomanometers and a stethoscope according to the recommendations of the Brazilian Society of Cardiology [21] at rest in a sitting position.

### 2.3. Biochemical Analyses

Serum concentrations of glucose, triglycerides, total cholesterol, and high-density lipoprotein cholesterol (HDL-c) were measured by an enzymatic colorimetric method using commercial kits (Labtest^®^, Lagoa Santa/MG, Brazil). The HbA1c was measured by immunoturbidimetric inhibition. All biochemical tests were performed using an automatic biochemical analyzer CMD 800i (Wiener Lab Group^®^, Rosario, Argentina). Serum insulin and C-peptide concentrations were measured by chemiluminescence using commercial kits (Abbott^®^, Abbott Park, IL, USA) and the automatic immunoassay analyzer Architect i1000SR (Abbott^®^, Abbott Park, IL, USA). The homeostasis model assessment (HOMA) was used to assess insulin sensitivity, based on C-peptide and glucose levels. Calculations were made using the HOMA calculator (University of Oxford, United Kingdom).

### 2.4. Food Intake

The 24-h dietary recalls over three days (including two weekdays and one weekend day) were obtained by Multiple-Pass method [22], and analyzed using the NutWin software (Department of Informatics, Paulista School of Medicine/UNIFESP, São Paulo/SP, Brazil), which includes the updated versions of the Brazilian Table of Food Composition (TACO), the Table of Nutritional Composition of Food Consumed in Brazil, the United States Department of Agriculture (USDA) Food Composition Databases, and food labels. The adequacy of usual dietary intake of nutrients by each group was evaluated according to the estimated average requirement (EAR) and adequate intake (AI) for potassium values proposed by the dietary reference intakes [23,24]. The probability of inadequate food intake by group was evaluated by z-score, which was calculated from the difference between the EAR corresponding to each nutrient and the mean ingestion of the group divided by the standard deviation of the intake. Potassium intake values were compared with AI values [24].

### 2.5. Statistical Analyses

The Kolmogorov–Smirnov test was used to evaluate data homogeneity. Non-homogeneous data were subjected to logarithmic transformation. The identification of individuals with inaccurate report of energy intake was performed using the methodology proposed by Mccrory et al. [25], based on the ratio between the reported energy intake and the total energy expenditure predicted, calculated by the equation of Vinken et al. [26] and considering the cut-off value of ± one standard deviation. The usual dietary intake was calculated using the Multiple Sources Method (MSM) (https://msm.dife.de/) [27]. Micronutrient intake values were adjusted for total energy intake according to residual method proposed by Willett et al. [28].

To verify associations between response variables, and to characterize individuals according to dietary intake of zinc, magnesium, calcium, and potassium, a hierarchical cluster analysis was used. From this analysis, the mean values corresponding to the energy-adjusted dietary intake of the four minerals were auto-scaled, and the similarities between the individuals calculated according to the Euclidean squared distance, while the Ward’s method was used for the formation of clusters. Differences between the two clusters formed was assessed using the Student’s *t*-test (for normal data distribution) or the Mann–Whitney test (for non-normal data distribution).

Regression models were used to assess the effect of the micronutrient intake on the glycemic control. Independent variables with *p* ≤ 0.20 in the bivariate analyses were included in the models using the enter method. *p* ≤ 0.20 was adopted according to the criterion of Wang et al. [29], established for the testing of a greater number of predictor variables in the model. Thus, a binary logistic regression model was applied using %HbA1c values above 7.0 [30] and cluster 1 (lower micronutrient intake) as risk variables. In addition, multiple regression models were applied to assess the effect of each micronutrient intake on %HbA1c separately. Multicollinearity was assessed by variance inflation factors (VIF) and no correlation was identified between the independent variables (VIF <10). Both regression models used sex, age, and time of T2DM diagnosis as adjustment variables.

The results were expressed as means and standard deviations, and as absolute and relative frequencies. A significance level of 5% was adopted for all tests. All statistical analyses were performed using SPSS for Windows Version 17.0 (SPSS Inc., Chicago, IL, USA).

## 3. Results

After exclusion of participants with missing dietary information, 95 individuals with T2DM were finally evaluated; 69.5% of them were female and mean age was 48.6 ± 8.4 years. Most individuals were overweight or obese (81.0%), and mean BMI and body fat percentage values were 30.22 ± 6.78 kg/m^2^ and 34.98 ± 7.94%, respectively. Waist circumference measurement showed 85.3% of the participants at increased risk of obesity-associated metabolic diseases (Table 1).

Deficient glycemic control was observed, as indicated by values of fasting glucose and %HbA1c above the cut-off point for disease control (fasting glucose < 154 mg/dL; %HbA1c < 7.0) [30]. Regarding lipid profile, 73.7% of the participants presented low HDL-c levels, and 37.9%, 10.5%, and 3.1% presented hypertriglyceridemia, mixed hyperlipidemia, and hypercholesterolemia, respectively.

**Figure 1:**
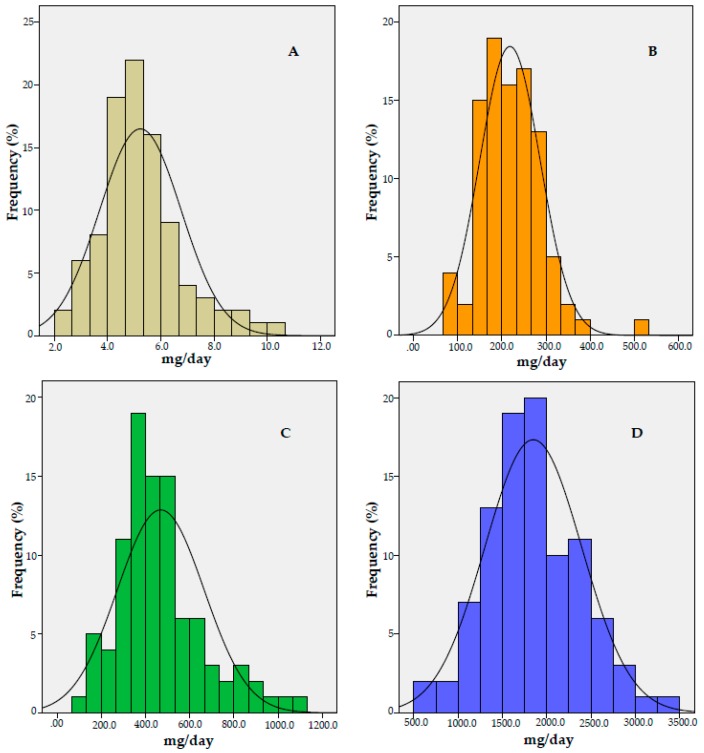
Histogram of energy-adjusted intake of zinc (**A**), magnesium (**B**), calcium (**C**), and potassium (**D**), and prevalence of inadequate intake in individuals with type 2 diabetes mellitus (T2DM) (*n* = 95). (**A**) Group intake (mean ± SD): 5.23 ± 1.53 mg/day; *Z*-score: 3.06 (men) and 0.94 (women). (**B**) Group intake (mean ± SD): 218.36 ± 68.13 mg/day; *Z*-score: 1.80 (men) and 0.67 (women). (**C**) Group intake (mean ± SD): 468.95 ± 195.3 mg/day; *Z*-score: 1.96 (group). (**D**) Group intake (mean ± SD): 1848.45 ± 543.43 mg/day. SD: Standard deviation.

Energy intake was found to be underreported by 84.2% of the individuals. A prevalence of inadequacy in energy-adjusted zinc intake of 99.9% in males and 82.6% in females was observed. The prevalence of inadequate energy-adjusted magnesium intake for males and females was 96.4% and 74.9%, respectively. The energy-adjusted calcium intake showed a prevalence of inadequacy of 95.5%. All the individuals evaluated had energy-adjusted potassium intake below the AI values (Figure 1).

When energy-adjusted micronutrient (zinc, potassium, calcium, and magnesium) intake was compared between clusters, the group with lower micronutrient intake (cluster 1) showed significantly higher %HbA1c (*p* = 0.006) and serum triglyceride concentration (*p* = 0.01) in comparison with the group with higher intake (cluster 2). No statistical differences were seen with regard to the other variables studied (Table 2).

Considering the significant difference in %HbA1c between clusters, a binary logistic regression model was applied in order to investigate the risk factors for increased %HbA1c (Table 3). We found that alterations in the %HbA1c were dependent of the time (in years) taken to diagnose T2DM (*p* = 0.005) and lower micronutrient intake (cluster 1) (*p* = 0.028).

Multiple regression analyses showed that, for every 1 g of potassium and 100 mg of magnesium ingested, there was 1% and 0.7% reduction in %HbA1c, respectively. These results were influenced by sex and time taken to diagnose T2DM (Table 4).

## 4. Discussion

After assessing the effect of the energy-adjusted dietary intake of zinc, potassium, calcium, and magnesium on the glycemic control of individuals with T2DM, we found a three-fold increased risk of high %HbA1c when dietary intake was reduced. In addition, potassium and magnesium intakes were predictive of %HbA1c decrease. It is noteworthy that most evaluated individuals were likely to have inadequate ingestion of these micronutrients.

The association between low micronutrient intake and deficient glycemic control is well known and poor intake of these micronutrients is widely documented in T2DM [2,3,12,31]. However, most studies looked at the intake of each micronutrient individually, and studies evaluating the concomitant intake thereof are scarce.

Epidemiological studies have shown a significant association between low daily zinc intake and risk of T2DM [10,18,32], and the risk can be reduced by 20% upon daily intake of more than 13 mg of zinc [33]. Kanoni et al. [34] found a significant correlation between zinc intake and fasting glucose values, where the daily intake of every 1 mg of zinc was able to reduce the glucose concentration by 0.02 mg/dL. The same study was included in a recent systematic review, which reported that the serum zinc concentration has been negatively correlated with %HbA1c and fasting glucose values in T2DM [16].

The role of zinc in the functioning and maintenance of pancreatic β-cell mass, insulin biosynthesis, and maturation of secretory granules is well documented [9,10]. In addition, zinc inhibits tyrosine phosphatases by stimulating auto phosphorylation of insulin receptors [14,16]. Moreover, it is essential in the redox mechanisms as a component of the superoxide dismutase enzyme, thus being helpful against the oxidative stress generated by hyperglycemia [10,14].

The role of magnesium in the glycemic control has been shown in experimental studies on rats with induced T2DM. Magnesium supplementation improved insulin secretion and sensitivity, lipid profile, and inflammatory status [35,36]. Morakinyo et al. [36] also showed that rats supplemented with magnesium improved GLUT4 translocation and, consequently, the metabolic control.

Previous evidence also points to an inverse association between magnesium intake and T2DM risk [13,37]. Hypomagnesemia and low magnesium intake are very prevalent in individuals diagnosed with T2DM, especially in those with poor glycemic control [12,31,38]. In addition, reduced plasma concentration of magnesium may lead to changes in the glycemic control, since the body distribution of this micronutrient has an impact on insulin secretion and action [12].

However, studies addressing the effect of magnesium supplementation on glycemic control are controversial. Some studies have shown positive effects of magnesium supplementation on blood glucose and %HbA1c lowering, and on the increased insulin sensitivity [8,39,40,41]. However, other studies did not observe such effects, which may be explained by the presence of normomagnesemia, time of supplementation, and reduced number of individuals assessed [42,43,44,45,46,47]. Two meta-analyses studies showed reduction of 0.56 mmol/L (95% confidence interval (CI) = −1.10; −0.01) [48] and 0.37 mmol/L (95% CI = −0.74; −0.00) [4], respectively, in the fasting glucose levels upon magnesium supplementation.

Magnesium is an important cofactor of enzymes involved in the glucose metabolism, in which it binds to an ATP molecule, yielding the Mg–ATP complex that acts in phosphate transfer reactions [4,12,37]. Thus, magnesium participates in the autophosphorylation of the β-subunit of insulin receptors, proliferation and maintenance of pancreatic β-cells, activity of tyrosine kinases, and stimulation of proteins and substrates of the insulin signaling cascade [12,13].

Enhanced intakes of calcium and potassium are also associated with reduced risk of T2DM [5,15], due to the combined action of these minerals in the process of insulin release [7]. Within the pancreatic β-cells, the intracellular ATP/ADP ratio that follows the glucose metabolism leads to the closure of ATP-sensitive potassium channels, and subsequent depolarization of the plasma membrane. Calcium channels are then opened, allowing the calcium influx to the cells and subsequent activation of exocytosis of the insulin granules [7,11].

The fundamental role of calcium in body weight control and, consequently, in the maintenance of insulin sensitivity in the adipose tissue, is observed in animal models. Dietary calcium induces suppression of calcitriol, thus reducing lipogenesis and increasing lipolysis. In addition, it increases the uncoupling protein-2 (UCP2) expression in the adipose tissue. UCP2 is responsible for the transport and oxidation of mitochondrial fatty acids, which reduces lipid storage and adiposity [49].

Few studies have assessed the effect of calcium intake on the glycemic control. In an eight-week study, calcium supplementation (1500 mg per day) improved the insulin sensitivity in individuals with T2DM [50]. However, insulin sensitivity was not changed after non-diabetic obese individuals underwent 24 weeks of supplementation with 1000 mg of calcium per day [49].

Furthermore, magnesium intake is likely to be a confounding factor as it is highly correlated with calcium metabolism as observed in studies addressing the association between intake of both minerals and the risk of T2DM [51,52]. A relationship between calcium and vitamin D intakes was also observed as the interaction of these nutrients leads to reduced fasting insulin and %HbA1c levels in individuals with T2DM [53].

The relation between potassium intake and variables of glycemic control in individuals with T2DM is not widely addressed in the literature. Both low potassium intake and reduced potassium concentration in serum were shown to be significantly associated with reduced insulin sensitivity and with a compensatory increase in insulin secretion [5], as well as with risk of T2DM [17,54].

Considering that the adoption of healthy dietary and body weight control patterns is fundamental for the T2DM management [1,6], a high rate of underreported energy intake is observed among individuals with T2DM in this study, and this can be the main reason for the high prevalence of overweight and obesity here documented. Underreporting is a commonly observed practice among obese individuals and can affect the estimation of nutrient intake, consequently impairing nutritional and disease assessments [55,56]. In addition, the analysis of food intake that relies on 24-hour recalls may have limitations, since this method is susceptible to inaccurate reports about both the food consumed and the size of its portions [25]. However, these limitations were mitigated after calculation of intra and interindividual variability, in combination with the adjustment for energy intake of nutrients.

Furthermore, food evaluation involves data of tables of food composition, and such tables can be different depending on where the food is produced. Therefore, similar to the present study, the use of region or country-specific food composition tables may help to correct this confounding factor.

## 5. Conclusions

Overall, studies evaluating the effect of the concomitant intake of micronutrients on variables of glycemic metabolism in individuals with T2DM are scarce, especially when it comes to zinc, potassium, calcium, and magnesium. This highlights the importance of our results for a better evaluation of usual food intake in this population, which is a simple and low-cost procedure, as %HbA1c is routinely measured in individuals with T2DM and it is the gold standard for glycemic control evaluation.

Therefore, inadequate concomitant intake of zinc, potassium, calcium, and magnesium is related to poor glycemic control in individuals with T2DM, and the intakes of magnesium and potassium are predictors of %HbA1c reduction. Further studies on dietary intake of individuals with T2DM and their relation with glucose metabolism are needed.

## Figures and Tables

**Table 1:** Clinical, biochemical and nutrient intake variables of individuals with type 2 diabetes mellitus (T2DM).

Variables	T2DM (*n* = 95)
Age, years	48.6 ± 8.4
Sex	
Men, *n* (%)	29 (30.5)
Women, *n* (%)	66 (69.5)
Time of T2DM diagnosis ^1^, years	7.3 ± 6.2
Insulin therapy ^2^, *n* (%)	21 (22.1)
Oral antidiabetic agents ^2^, *n* (%)	70 (73.7)
Lipid-lowering agents ^2^, *n* (%)	24 (25.3)
Antihypertensive agents ^2^, *n* (%)	46 (48.4)
Weight, kg	78.9 ± 19.3
BMI, kg/m^2^	30.2 ± 6.8
Fat mass ^2^, %	35.0 ± 7.9
Waist circumference, cm	99.8 ± 14.3
Men, *n* (%)	
<94 cm	8 (27.6)
≥94 cm	21 (72.4)
Women, *n* (%)	
<80 cm	6 (9.1)
≥80 cm	60 (90.9)
SBP ^3^, mmHg	129.2 ± 17.0
DBP ^3^, mmHg	83.5 ± 15.3
Fasting glucose, mg/dL	180.1 ± 84.1
%Hb1Ac	8.1 ± 2.1
Insulin, µU/mL	13.8 ± 13.5
C-peptide, ng/mL	2.7 ± 0.8
HOMA2-%B	77.0 ± 56.2
HOMA2-%S	42.8 ± 20.9
HOMA2-IR	3.3 ± 2.9
Total cholesterol, mg/dL	193.4 ± 47.3
HDL-c, mg/dL	41.1 ± 10.4
LDL-c, mg/dL	117.4 ± 39.8
Triglycerides, mg/dL	174.3 ± 117.1
Energy intake, kcal/day	1469.4 ± 478.5
Protein intake, g/day	80.0 ± 20.2
Carbohydrate intake, g/day	210.1 ± 73.6
Lipid intake, g/day	37.1 ± 15.6
Zinc intake, mg/day	5.2 ± 1.5
Potassium intake, mg/day	1848.5 ± 543.4
Calcium intake, mg/day	469.0 ± 195.3
Magnesium intake mg/day	218.4 ± 68.1

Results presented in mean ± standard deviation and absolute frequency; ^1^ *n* = 91; ^2^ *n* = 93; ^3^ *n* = 89. %HbA1c: glycated hemoglobin percentage; BMI: body mass index; DBP: diastolic blood pressure; HDL-c: high-density lipoprotein cholesterol; HOMA: Homeostasis Assessment Model; LDL-c: low-density lipoprotein cholesterol; T2DM: type 2 diabetes mellitus; SBP: systolic blood pressure. Micronutrients adjusted for total energy intake using the residual method [28].

**Table 2:** Clinical, biochemical and nutrient intake variables of individuals with type 2 diabetes mellitus (T2DM).

Variables	*Cluster* 1 (*n* = 65)	*Cluster* 2 (*n* = 30)	*p*-value
Age, years	48.6 ± 8.6	48.9 ± 7.9	0.866 ^¥^
Time of T2DM diagnosis, years	7.2 ± 6.4	7.4 ± 6.0	0.909
BMI, kg/m^2^	30.4 ± 6.9	29.9 ± 6.7	0.762 ^¥^
Waist circumference, cm	100.8 ± 13.7	97.7 ± 15.5	0.332
Fat mass, %	34.6 ± 8.0	35.8 ± 7.8	0.483
Total cholesterol, mg/dL	198.8 ± 49.7	181.5 ± 40.0	0.097
HDL-c, mg/dL	41.9 ± 10.5	39.2 ± 10.3	0.247
LDL-c, mg/dL	117.9 ± 42.2	116.4 ± 34.5	0.867
Triglycerides, mg/dL	194.9 ± 131.1	129.6 ± 58.5	0.010
%Hb1Ac	8.3 ± 2.1	7.2 ± 1.7	0.006
C-peptide, ng/mL	2.7 ± 0.9	2.7 ± 0.7	0.963
Fasting glucose, mg/dL	190.3 ± 88.2	158.1 ± 70.7	0.082 ^¥^
Insulin, µU/mL	13.2 ± 11.7	15.1 ± 16.8	0.525
HOMA2-%B	73.7 ± 59.4	84.0 ± 48.8	0.407 ^¥^
Energy intake, kcal/day	1452.3 ± 471.4	1506.3 ± 499.7	0.611
Lipid intake, g/day	37.4 ± 15.1	36.5 ± 16.8	0.796
Protein intake, g/day	80.2 ± 19.7	79.4 ± 21.4	0.856
Carbohydrate intake, g/day	203.6 ± 74.1	224.2 ± 71.7	0.207
Zinc intake, mg/day	4.8 ± 1.4	6.1 ± 1.5	<0.001
Potassium intake, mg/day	1556.5 ± 344.3	2480.9 ± 301.4	<0.001
Calcium intake, mg/day	400.6 ± 136.4	616.9 ± 222.4	<0.001
Magnesium intake mg/day	191.5 ± 54.2	276.6 ± 58.5	<0.001

Results presented in mean ± standard deviation. *p*-value <0.05 was considered statistically significant. Student’s *t*-test for independent samples and ^¥^ Mann–Whitney test. %HbA1c: glycated hemoglobin percentage; BMI: body mass index; HDL-c: high-density lipoprotein cholesterol; HOMA: Homeostasis Assessment Model; LDL-c: low-density lipoprotein cholesterol; T2DM: type 2 diabetes mellitus. Micronutrients adjusted for total energy intake using the residual method [28].

**Table 3:** Binary logistic regression model of glycated hemoglobin percentage (%HbA1c) and clusters formed from energy-adjusted mineral intake by individuals with type 2 diabetes mellitus (T2DM).

Dependent Variable	Covariables	OR (95% CI)	*p*-value
%Hb1Ac ^§^	Age (years) ^£^	0.987 (0.931; 1.046)	0.661
	Sex ^§^	1.598 (0.580; 4.405)	0.365
	Time of T2DM diagnosis (years) ^£^	1.155 (1.043; 1.278)	0.005
	Cluster 1 ^§^	3.041 (1.131; 8.175)	0.028

*p*-value <0.05 was considered significant. *p*-value of model: 0.003 and *r*^2^: 0.221. Hosmer and Lemeshow test: 0.591. ^§^ Variables included in the model in the dichotomous format. ^£^ Variables included in the model in continuous format. Model adjusted by sex, age, and time of T2DM diagnosis. %HbA1c: glycated hemoglobin percentage; CI: confidence interval; OR: odds ratio; T2DM: type 2 diabetes mellitus. Risk classification used %HbA1c >7% [30] and cluster 1 with lower combined intake of zinc, potassium, calcium, and magnesium.

**Table 4:** Multiple linear regression models of glycated hemoglobin percentage (%HbA1c; dependent variable) and energy-adjusted mineral intake by individuals with type 2 diabetes mellitus (T2DM).

	Independent Variables	β (95%CI)	*p*-value	*r*^2^ Adjusted
Model 1 ^1^	Age (years)	−0.032 (−0.082; 0.019)	0.216	0.143
	Sex ^§^	1.069 (0.209; 1.930)	0.015	
	Time of T2DM diagnosis (years)	0.117 (0.051; 0.183)	0.001	
	Zinc intake (mg/day)	−0.017 (−0.283; 0.250)	0.902	
Model 2 ^2^	Age (years)	−0.028 (−0.077; 0.021)	0.259	0.198
	Sex ^§^	0.925 (0.086; 1.765)	0.031	
	Time of T2DM diagnosis (years)	1.118 (0.054; 0.182)	<0.001	
	Potassium intake (mg/day)	−0.001 (−0.002; 0.000)	0.017	
Model 3 ^2^	Age (years)	−0.029 (−0.079; 0.021)	0.253	0.151
	Sex ^§^	1.025 (0.163; 1.887)	0.020	
	Time of T2DM diagnosis (years)	0.119 (0.053; 0.184)	0.001	
	Calcium intake (mg/day)	−0.001 (−0.003; 0.001)	0.377	
Model 4 ^2^	Age (years)	−0.031 (0.080; 0.017)	0.206	0.201
	Sex ^§^	1.009 (0.177; 1.840)	0.018	
	Time of T2DM diagnosis (years)	0.117 (0.053; 0.181)	<0.001	
	Magnesium intake (mg/day)	−0.007 (−0.012; −0.001)	0.015	

^1^ *p*-value of model: 0.002. ^2^ *p*-value of model: <0.001. β non-standardized. Variables included in the models in the continuous format and in the ^§^ dichotomous format. All models adjusted by sex, age and time of T2DM diagnosis. %HbA1c: glycated hemoglobin percentage; CI: confidence interval; T2DM: type 2 diabetes mellitus.
